# Model for ethical triaging of electroconvulsive therapy patients during the COVID-19 pandemic

**DOI:** 10.1192/bjb.2020.99

**Published:** 2020-08-19

**Authors:** Phern-Chern Tor, Jacinta Tan, Colleen Loo

**Affiliations:** 1 Institute of Mental Health, Singapore; 2 Aneurin Bevan University Health Board, Newport, UK; 3 University of New South Wales, Sydney, Australia; 4 The Black Dog Institute, Sydney, Australia; 5 St George Hospital, Sydney, Australia

**Keywords:** Electroconvulsive therapy, ethics, depressive disorders, suicide, stigma and discrimination

## Abstract

Electroconvulsive therapy (ECT) is an essential treatment for severe mental illnesses such as depression with suicidality and catatonia. However, its availability is being threatened by resource limitations and infection concerns due to the COVID-19 pandemic. This may necessitate the triage of patients for ECT but there is no established ethical framework to prioritise patients. We offer an application of an ethical framework for use of scare medical resources in the ECT setting.

Electroconvulsive therapy (ECT) is the most effective acute treatment for severe depression.^1^ It is also effective in reducing psychotic symptoms in treatment-resistant schizophrenia and in treating mania and catatonia. The National Institute for Health and Care Excellence (NICE) treatment guidelines for ECT^2^ and the Royal College of Psychiatrists’ position statement on ECT^3^ state that ECT should be a first-line treatment where rapid response is required because of high suicide risk, poor oral intake or other conditions where the patient's physical health is at risk. These guidelines also state that valid informed consent should be obtained without pressure or coercion, in the context of significant stigma, discrimination and controversy associated with the treatment.^2^ A substitute decision maker should be available to patients lacking capacity to give consent, as there is increasing evidence that patients lacking capacity have equivalent^4^ to superior responses^5^ to ECT compared with capacitous patients. Despite its impressive effectiveness and broad spectrum of effect, ECT has experienced at least 20 years of decreasing in-patient use in the USA. In 2017 a study reported that only 1 in 10 US hospitals offered ECT and only 1.5% of severely depressed in-patients received ECT,^6^ the most effective treatment for severe depression. These trends are also evident in the UK and Ireland.^7^ Furthermore, the use of ECT is controversial and has its detractors and some consider it unacceptable in modern psychiatry.^8^

With the COVID-19 pandemic, ECT challenges have compounded from a problem of getting patients to accept ECT to an additional challenge of struggling to continue providing ECT for existing patients. Already scarce ECT resources have been further reduced by lack of personal protective equipment (PPE), restriction of anaesthesia and limited institutional support.^9^ The International Society of ECT and Neurostimulation (ISEN) has published a position statement on ECT during COVID-19 which includes classifying cases into elective, urgent/essential and emergency and suggests triaging patients to reduce demand for ECT.^10^ This recommendation to triage patients is a relatively novel situation for many ECT practitioners, who are more accustomed to a ‘first come, first served’ situation in routine ECT practice. We suggest a useful ethical model that can be used in conjunction with existing ethical frameworks to assist ECT practitioners to take a consistent approach to triaging patients for ECT, rather than relying on individual institutional norms or clinician intuition.

## General medical ethics applied to ECT

A commonly accepted framework for medical ethics uses the ‘Georgetown principles’ of beneficence, non-maleficence, autonomy and justice. Ottosson & Fink^11^ suggest the following ECT-specific considerations for each principle.

### Beneficence

The highest priority for ECT should be patients who would gain the most potential benefit from the treatment (e.g. those with psychoses and involuntarily committed or with depression with high suicidality), have the potential for fast response (e.g. catatonia) and have the highest risk to life or long-term disability.

### Non-maleficence

Given that mortality with ECT is lower than overall mortality associated with general anaesthesia,^12^ and lower than if the patient had not received ECT,^13^ the main side-effect of concern is cognitive impairment. However, the cognitive impairment is often transient, lasting for a shorter period than the therapeutic effect of ECT and can be minimised using empirically derived dosing of ECT.^14^

### Autonomy

ECT ideally should be administered with the patient's consent. However, no patient should be denied ECT just because they lack capacity to consent to treatment. Ottosson & Fink make a distinction between weak paternalism in the patient's best interests and authoritarianism that discounts the patient's autonomy. Prescribing ECT with a substitute decision-making process or in patient's best interests is increasingly supported by recent evidence of equivalent to superior outcomes in patients lacking capacity to consent to ECT.^5^

### Justice

There are three broad forms of distributive justice: egalitarian (equal access), libertarian (the right to social and economic liberty) and utilitarian (maximise public utility). During a time of limited resources, Emanuel *et al*^15^ argue that a utilitarian approach is the most appropriate, i.e. emphasising population outcomes by triaging patients who are most likely to respond and derive the most benefit from ECT with the least risk of harm to the patients and the ECT team. This could even mean pausing ECT for a patient with low utilitarian potential in order to start ECT for a patient with high utilitarian value.

For the purposes of this discussion, the context is that of a predominantly public or taxpayer-funded healthcare system rather than one that is predominantly insurance or self-funded. The former system is more likely to face the problems of scare resources requiring healthcare rationing^16^ and the libertarian aspects of justice may be less dominant.

## What has changed for ECT during COVID-19?

Beyond decreased ECT availability, there are at least five other factors to take into account when considering ECT during COVID-19:

- disruption of routine care delivery during the crisis, leading to increased risk of harm to patients’ health
- increased risk of patients getting COVID-19 owing to lack of ECT (e.g. delayed discharge from hospital while their illness resolves more slowly and higher vulnerability to COVID-19 among severely mentally ill patients^9^)
- increased risk of patients getting COVID-19 while attending for ECT (e.g. repeated out-patient appointments for ECT, requiring patients to travel more frequently)
- increased risk to the team delivering ECT, due to the higher risk of infection from patients receiving general anaesthesia and potential aerosolisation of patients’ respiratory material
- utilisation of highly skilled staff during a time of scarcity, in particular the services of anaesthetists, who could otherwise be redeployed running intensive treatment units (ITUs).

## How should we ethically triage ECT patients during COVID-19?

Emanuel et al^15^ provide a four-point framework to guide rationing of scarce healthcare resources during COVID-19: (a) maximise benefits; (b) treat people equally; (c) promote and reward instrumental value; and (d) give priority to the worst off.

Maximising benefits is achieved by prioritising limited resources for saving the most lives and with maximal improvement in patients’ lives after treatment. Treating people equally refers to not letting a patient's financial resources or status affect treatment allocation. Promoting and rewarding instrumental value is giving priority to those who can save or have saved others. Lastly, giving priority to the worst off could be interpreted as giving priority to the sickest or to younger people, who would have lived the shortest lives if untreated. Table 1 lists Emanuel et al's principles, with a column added describing how they could be applied to an ECT setting.

**Table 1:** Ethical values to guide rationing of scarce healthcare resources in the COVID-19 pandemic, adapted for electroconvulsive therapy (ECT)^a^

Ethical values and guiding principles	Application to COVID-19 pandemic	Specific ECT applications
Maximise benefits		
Save the most lives	Receives the highest priority	Prioritise in-patients with severe psychotic depression, lethal catatonia, neuroleptic malignant syndrome, manic delirium Deprioritise patients with predictors of poorer outcome to ECT (e.g. personality disorder, depression that is more chronic or treatment resistant, without suicidality or dangerousness) Deprioritise patients with high medical risk during ECT Deprioritise patients who must expose themselves to greater risk of COVID-19 infection to access ECT (e.g. living far from the ECT facility) Give higher-dose ECT and avoid milder ECT modalities, to minimise number of sessions and patient and staff risk of exposure to COVID-19
Save the most life-years – maximise prognosis	Receives the highest priority	
Treat people equally		
First come, first served	Should not be used	Use random allocation to prioritise patients with similar prognosis
Random selection	Used for selecting among patients with similar prognosis	
Promote and reward instrumental value (benefit to others)		
Retrospective – priority to those who have made relevant contributions	Gives priority to research participants and healthcare workers when other factors, such as maximising benefits, are equal	Prioritise patients who are healthcare workers or work in essential services Deprioritise patients who pose a higher risk of infecting the ECT team, to conserve ECT resources
Prospective – priority to those who are likely to make relevant contributions	Gives priority to healthcare workers	
Give priority to the worst off		
Sickest first	Used when it aligns with maximising benefits	Prioritise younger premorbidly well patients with acute onset of an ECT-responsive psychiatric disorder
Youngest first	Used when it aligns with maximising benefits such as preventing spread of the virus	

a. Based on Emanuel et al's four-point framework.^13^

## Applying these principles to clinical scenarios

Applying Emanuel et al's principles and the ECT-specific considerations outlined above, a high-priority patient might be a young healthcare worker in your healthcare institution with no psychiatric history and admitted for an acute onset of psychotic depression, catatonic symptoms and a serious suicide attempt, whose family is supportive of ECT. Two clinical scenarios are presented for further discussion.

### Scenario 1

A 33-year-old labourer with a long history of well-controlled schizophrenia is brought to the psychiatric emergency room with symptoms suggestive of acute onset of stuporous catatonia (mutism, negativism, posturing) and poor oral intake for 2 weeks. His BMI is 16, he is clinically dehydrated and his blood pressure is borderline hypotensive. Although he has no clear symptoms of COVID-19, he lives in a large accommodation facility with dozens of people who have tested positive for COVID-19. The facility already follows recommended infection control procedures and screening, instituted several weeks before this presentation.

This is a challenging clinical scenario where there is a psychiatric emergency (catatonia with poor oral intake) that is highly responsive to ECT, but in a patient with a primary psychiatric condition (schizophrenia) that may not be indicated for ECT as a first-line treatment and moderate to high risk of having COVID-19. Using the proposed ethical framework below, the patient's youth and catatonia would satisfy the principles of ‘maximising benefits’ and ‘giving priority to the worst off’, but it would be contrary to the principle of ‘promoting and rewarding instrumental value’, as treating the patient would expose the ECT team and other patients to a significant risk of getting COVID-19, especially if the ECT unit is not fully prepared to deal with suspected or positive COVID-19 patients.

As with many ethical scenarios, the initial approach to resolution would be a medical solution. If the patient's catatonia responds to high-dose benzodiazepines (e.g. lorazepam), then there would be no need to consider the use of ECT. If benzodiazepine treatment failed, a negative result on polymerase chain reaction testing for COVID-19, the lack of other patients requiring ECT and the availability of specialised treatment facilities (e.g. negative-pressure rooms) might mitigate the risk of infection of ECT team members and allow ECT to proceed in an ethical fashion.

### Scenario 2

Another challenging scenario is that of a 67-year-old woman who is admitted to a psychiatric ward for the in-patient treatment of major depressive disorder with acute suicidality. She also has a history of borderline personality disorder, comorbid generalised anxiety disorder and panic disorder and has not previously experienced much response to full courses of psychotherapy and adequate pharmacotherapy. She consented to a course of ECT and had already received five sessions before a fellow ward patient was diagnosed with COVID-19. The entire ward is quarantined as a result. The patient is keen to continue her ECT course as she has not yet felt any improvement and other treatment options have been relatively ineffective.

This scenario has a patient with both positive (depression, older age) and negative (history of personality disorder and anxiety) predictors for ECT response,^17^ no response to the first five ECT treatments and a significant risk of having presymptomatic COVID-19. The principle of ‘maximizing benefits’ is less clear here, as her prognosis of responding to ECT is mixed, and the principle of ‘promoting and rewarding instrumental value’ would discourage continued ECT, at least until she is cleared of COVID-19. The other two ethical principles, of ‘treating people equally’ and ‘giving priority to the worst off’, may be useful to help clarify the ethical position. The former might suggest that other patients in the same ward who are also receiving ECT with similar prognosis would have an equivalent claim for ECT and the patient's request for continued ECT should not give her higher priority. The latter would further refine this point by considering the severity of the woman's psychiatric diagnosis and perhaps giving sicker patients priority to ECT (e.g. a patient with severe psychotic depression, who is also highly likely to respond to ECT).

## What is the road forward for ethical triaging for ECT during COVID-19?

Where treatment resources are limited, fair allocation of resources requires careful consideration of all relevant ethical issues in the context of the local resources and situation. The ideal solution is to ensure adequate ECT resources, so that both high- and low-priority patients can receive high-quality ECT. This requires deliberate short- and long-term planning and negotiation for scarce resources within healthcare systems, the exploration of new ECT resources (e.g. advanced practice nurses for both anaesthesia and ECT delivery,^18^ dedicated ECT suites to avoid competition with surgical needs) and adequate PPE for ECT staff and patients. The current COVID-19 crisis has placed significant strain on healthcare resources for many months, and at the height of the pandemic many non-emergency non-COVID services were suspended to divert resources to deal with the COVID-19 emergency. This has resulted in a significant backlog of untreated patients, with consequent increased pressure on already scarce resources. Furthermore, as healthcare systems reorient themselves to provide routine care and begin to deal with the backlog, there remains a need to maintain social distancing and scrupulous hygiene, for instance deep cleaning operating theatres and equipment between each patient, which will reduce efficiency and capacity. For all these reasons, these pressing ethical dilemmas about how to prioritise patients must be addressed to ensure that patients with non-COVID disorders continue to have their healthcare needs met fairly and equitably in a fully accountable way. These efforts should be a priority even after the COVID-19 situation eventually resolves. Given the SARS outbreak in 2003^19^ and the current COVID-19 outbreak,^9^ which both caught most of the world largely unprepared, there is a strong ethical imperative to prepare for the future third coronavirus outbreak or, indeed, second or third waves of COVID-19 either locally or globally.

## About the authors

**Phern-Chern Tor**, MBBS, DFD(CAW), MMed(Psych), FAMS, is a consultant psychiatrist and head of the Neurostimulation Service at the Institute of Mental Health, Singapore. **Jacinta Tan**, MBBS, MA, MSc, DPhil, FRCPsych, is a consultant child and adolescent psychiatrist at the Specialist Child and Adolescent Mental Health Services, Aneurin Bevan University Health Board, Newport, UK. **Colleen Loo**, MBBS, MD, FRANZCP, is Professor of Psychiatry in the School of Psychiatry, University of New South Wales, Sydney; Professorial Fellow at The Black Dog Institute, Sydney; and a psychiatrist and clinical academic based St George Hospital, Sydney, Australia.
